# Does physical activity affect social skills and antisocial behavior? The gender and only child status differences

**DOI:** 10.3389/fpubh.2024.1502998

**Published:** 2024-11-18

**Authors:** Yuke Yang, Yan Gao, Xiangren Yi, Yining Hu, Liangyu Zhao, Lu Chen, Wenze Sui, Shuoqin Zhang, Sen Ma

**Affiliations:** School of Physical Education, Shandong University, Jinan, China

**Keywords:** physical activity, social skills, antisocial behavior, Chinese adolescents, fixed effects model, only child status, gender difference

## Abstract

**Background:**

This study aims to explore the effects of physical activity on adolescents' social skills and antisocial behavior, as well as gender and only child status differences among Chinese adolescents.

**Methods:**

We used longitudinal data, collecting baseline data for 2021–2022 and follow-up in 2022–2023. A total of 3,206 students were enrolled, including 1,510 males (Age ± SE: 13.62 ± 1.69) and 1,696 females (Age ± SE: 14.09 ± 1.85), 1,339 only children (Age ± SE: 13.76 ± 1.95), and 1,867 non-only children (Age ± SE: 13.95 ± 1.66). The fixed effects model was used to identify the effects of different types of physical activity on adolescent social behavior and the heterogeneity analysis.

**Results:**

(1) We found that skipping rope (β = 2.284, t = 5.76), walking (β = 3.495, t = 9.53), cycling (β = 1.271, t = 3.21), jogging (β = 2.614, t = 5.92), and badminton (β = 1.409, t = 2.96) had a positive impact on adolescents' social skills. Tag games (β = −1.615, t = −3.83), swimming (β = −2.862, t = −4.42), dancing (β = −1.11, t = −2.29), and skiing (β = −2.771, t = −3.27) had a negative impact on adolescents' social skills. Skipping rope (β = −1.596, t = −5.86), walking (β = −1.814, t = −7.44), cycling (β = −1.066, t = −4.07), and jogging (β = −1.617, t = −5.30) tended to reduce adolescents' antisocial behavior. Tag games (β = 1.685, t = 5.54), swimming (β = 0.947, t = 2.06), ice skating (β = 1.772, t = 2.71), and skiing (β = 1.468, t = 2.31) tended to increase adolescents' antisocial behavior. In addition, we found that these effects differ by gender and only child status. (2) Boys had significantly higher participation rates in activities. Only children had higher participation rates in skipping rope, baseball or softball, and volleyball, but non-only children had higher participation rates in walking, cycling, jogging, other aerobic exercises, and badminton. (3) Girls had higher social skills, but boys and only children had significantly higher rates of antisocial behavior.

**Conclusion:**

Our results provide a novel understanding that can inform interventions and improvements in social behavior among Chinese adolescents. Stakeholders such as educators, policymakers, and health professionals can develop more tailored and effective strategies to promote positive social development among adolescents.

## 1 Introduction

Adolescence is a uniquely transformative period in life, during which cognition, emotion, and social functions continuously evolve and gradually take shape (1). Adolescence, a time marked by a strong desire for acceptance by peers and a fear of social exclusion (2), often brings heightened social needs. Therefore, fostering social skills in adolescents is crucial for the development of healthy social networks and their overall wellbeing. However, schools present adolescents with complex social settings (3). These circumstances pose severe challenges to the shaping and formation of their mental health, academic performance, social skills, and societal behavior.

Social skills are the important abilities that students need to succeed and be happy in school, with peers, and in their daily interactions with family members (4). It is the positive responses that students generate in social interactions, such as cooperation, self-confidence, responsibility, empathy, self-control, and communication (5). These skills can prevent negative emotions and behaviors like antisocial actions and aggression (3). In contrast to social skills, antisocial behavior (ASB) is defined as behavior that is illegal but does not necessarily result in prosecution (6). ASB typically manifests as impulsivity and destructiveness (7), with impulsivity referring to actions that cannot be inhibited and destructiveness to deliberate harm or annoyance to others (8). ASB in adolescents are often linked to societal dangers such as school bullying (9) and criminal activities (10).

Physical activity (PA) is widely recognized as a determinative factor in promoting adolescent health, effectively reducing negative emotions in the face of stress (11). The UK Chief Medical Officer's PA guidelines suggest that all adolescents engage in at least 60 min of moderate to vigorous PA daily (12). Studies have confirmed the significant impact of PA on children's social skill levels (13). Different types of PA have varied effects on adolescents, with aerobic exercises improving mental health and cognitive function, strength training alleviating depressive symptoms, and team sports enhancing social skills (14), such as football (15). Activities like swimming, cycling, and sports games can improve social skills, communication, and expression in children with autism (16, 17). However, some scholars have reported that while PA interventions have a significant positive effect on the social outcomes of at-risk adolescents, there is a high risk of bias in these studies (18). To our knowledge, the impact of different types of exercise on the social skills and ASB of Chinese adolescents is yet to be determined.

According to affective attribution theory, in social interactions, males are more likely to attribute negative emotions to external factors, thereby releasing their emotions, while females tend to attribute them to internal factors, leading to a greater propensity for self-imposed isolation (19). This has resulted in boys being more prone to ASB (20). Girls, on the other hand, possess stronger interpersonal skills and are more likely to engage in prosocial behaviors such as cooperation, demonstrating better abilities in peer interactions (21). However, they also have more sensitive interiors and face greater interpersonal pressure (21), which can lead to more severe internalized symptoms and greater distress when experiencing adverse events or social exclusion (22, 23). Scholars have confirmed that although girls are more socially adept and engage in more social activities, boys prefer to participate in PA (24). There are also significant gender differences in adolescent PA (25, 26). Other scholars have found that PA boosts social skills significantly more in girls than in boys (13). Therefore, considering the potential impact of gender on outcomes, it is necessary to incorporate gender differences into research.

According to the limited attention theory, an individual's cognitive capacity is finite, attention to one thing necessarily reduces attention to others during the allocation of resources (27). Therefore, there are significant differences between only children and non-only children in terms of age, urbanization, family income, and family environment (28). Family environment is a crucial factor in shaping adolescent PA and social behavior (28). The participation of adolescents in PA is inseparable from the support of family, friends, and peers (29). Siblings have a closer relationship with adolescents than their peers and friends, providing a continuous and enduring presence in their lives, and adolescents with siblings are more easily influenced in various ways (30). This also brings about differences in social behavior and PA preferences for only child status. On the one hand, non-only children have higher levels of PA participation, with siblings encouraging their involvement in sports and establishing healthy behavior patterns (31). On the other hand, only children and non-only children are in different social networks, leading to different human, material resources, and interpersonal relationships, which in turn affect their social behavior (32). Therefore, we believe that exploring the only-child status of Chinese adolescents will help to further understand the intrinsic mechanisms of the impact of PA on social skills and ASB.

Existing research does not provide a consistent, authoritative account of the impact of different PA on the social skills and ASB of Chinese adolescents, nor does it include heterogeneity analysis for this population regarding gender and only-child status. Therefore, on the basis of studying the relationship between these factors, verifying whether gender and only-child status have a potential impact on this relationship will not only help deepen our understanding of the PA and social behavior of Chinese adolescents but also draw attention to PA that can be used to intervene in adolescent social behavior. Our findings will provide new evidence and insights for intervening and improving the social skills and ASB of Chinese adolescents.

## 2 Materials and methods

### 2.1 Participants

This longitudinal study was conducted in accordance with the guidelines set forth in the Helsinki Declaration. All procedures involving human subjects were approved by the Ethics Committee of Shandong University (20180517). Prior to the initiation of the survey, informed consent forms were completed by both parents and students. Two waves of data were collected from 186 junior and senior high schools across 17 cities in Shandong Province, China, during the 2021–2022 and 2022–2023 academic years using population proportionate sampling (PPS) method. All staff involved in data collection and processing underwent two standardized trainings. During the survey, trained investigators organized students to measure physical fitness using standardized guidelines and guided them in completing online questionnaires. All data were collected voluntarily, anonymously, and confidentially, and the collected data were stored on a password-protected website.

A total of 17,084 baseline samples were collected in 2021–2022, and 16,494 follow-up samples in 2022–2023. After matching and merging the data from the 2 years, 3,705 samples remained. After excluding samples with missing age and gender information, a final effective sample of 3,206 was obtained. This included 1,510 males (age: 13.62 ± 1.69), 1,696 females (age: 14.09 ± 1.85), 1,339 only children (age: 13.76 ± 1.95), and 1,867 non-only children (age: 13.95 ± 1.66).

### 2.2 Measures

#### 2.2.1 Physical activity

PA was measured using the Physical Activity Questionnaire for Older Children (PAQ-C), a self-administered 7-day recall tool designed to assess the general PA levels of students aged 8 to 14 during school days (33). For the purpose of this study, the first item of the PAQ-C was selected to collect data on different types of PA engaged in by adolescents. In line with the actual participation patterns of Chinese adolescents in PA, the types of activities collected included: skipping rope, roller skating, tag games, walking, cycling, jogging, other aerobic (e.g., mountain climbing), swimming, baseball or softball, dance-related activities, badminton, skateboarding, football, volleyball, basketball, ice skating, and skiing. All adolescents were asked to recall their participation in these PA over the past week and select the corresponding options. No participation in a week was scored as 1 point, while participation seven times or more was scored as 5 points. Each item was scored on a 5-point scale, with a range of 1–5 points, where higher scores indicated a higher frequency of participation. Using baseline data, exploratory factor analysis was conducted to examine the construct validity of the scale (KMO = 0.952, *P* = 0.000), and Cronbach's alpha was used to test its reliability (Cronbach's alpha = 0.924). The results indicated good reliability and validity, suggesting that it was suitable to extract information from this scale.

#### 2.2.2 Social skills and antisocial behavior

Adolescents' social skills and ASB were measured using the School Social Behavior Scale (SSBS) (34). The scale consists of 65 items divided into two dimensions: social skills and ASB. The SSBS was designed to assess social skills and ASB related to both teachers and peers. Social skills measured adolescents' learning abilities, self-management skills, and interpersonal communication skills. ASB was measured in terms of Hostile-Irritable, Antisocial-Aggressive, and Disruptive-Demanding. All items are scored on a five-point scale, ranging from “never” to “often”, corresponding to scores of 1–5. The total score for social skills and ASB was calculated by adding up the scores of all items, with higher scores indicating a higher frequency of occurrence. The social skills scale was positively predictive, while the ASB scale was inversely predictive. Higher scores in social skills and lower scores in ASB represent better adaptation to the school environment. The reliability and validity of both the social skills and ASB scales were confirmed. Specifically, the reliability of the social skills subscale was Cronbach's alpha = 0.970, with validity KMO = 0.985, *P* = 0.000. The reliability of the ASB subscale was Cronbach's alpha = 0.968, with validity KMO = 0.986, *P* = 0.000.

#### 2.2.3 Covariates

We also measured control variables that might affect the outcomes, which include gender, age, only-child status, and socioeconomic status (SES). Gender was represented by two dummy variables (1 = Male; 2 = Female). Whether the child is an only child was categorized into two types (1 = Yes; 2 = No). SES consists of five items, which include the educational level of both parents (with nine options ranging from no education = 1, primary school = 2, junior high school = 3, vocational school = 4, vocational high school = 5, high school = 6, associate degree = 7, bachelor's degree = 8, graduate and above = 9), the occupation of both parents (with 12 options including unemployed, laid off, farmer, self-employed, general employee in commerce and service, general worker, skilled worker, private enterprise owner, ordinary clerk, ordinary assistant, technician, teacher, engineer, doctor, lawyer, or other professional, senior management in enterprise or company, leader or department head in national organs or institutions), and self-assessment of family economic status (ranging from low to high with scores from 1 to 5, with higher scores indicating better self-assessed family economic status). The total score for SES was calculated by adding the scores of the five items, with higher scores representing a better SES.

### 2.3 Data analysis

In this study, to eliminate unobserved heterogeneity at the individual level, we used 2 years of data for longitudinal estimation and heterogeneity analysis. First, we conducted descriptive analysis of the sample using independent samples *t*-tests and validated the differences in types of PA, social skills, and ASB by gender and only-child status. Next, the Hausman test was used to determine whether the fixed effects (FE) or random effects (RE) model should be employed. The results of the Hausman test rejected the null hypothesis, indicating that the FE was more suitable for this study. Furthermore, the FE model validated the relationship between the explanatory variables—different types of PA—and the explained variables—social skills and ASB. Lastly, to test the robustness of the study, the paper further enhanced the robustness of the results by validating Robust t-statistics. Heterogeneity was tested by conducting separate analyses for only-child status, gender, and different types of PA. To assess multicollinearity among the variables, we calculated the Variance Inflation Factor (VIF), which had a mean of 2.8, indicating no collinearity issue since it is less than the threshold of 5. Additionally, the FE model used in this study mitigated potential endogeneity problems.

During the analysis process, SPSS 27.0 was used for descriptive analysis of the data, while Stata 17.0 was employed for the Hausman test, VIF test, heterogeneity test, and FE.

## 3 Results

### 3.1 Descriptive analysis

Tables 1, 2 present the characteristics of participants in the first wave of data (2021–2022). Out of the 3,206 participants, there were 1,510 males and 1,696 females, with an average age of 13.62 ± 1.69 for males and 14.09 ± 1.85 for females. There were 1,339 only children and 1,867 non-only children. The average educational level of the participants' parents was junior high school and above. Additionally, we found significant gender differences in age, father's educational level, father's occupation, and SES. Girls were older than boys on average. Boys had a lower SES compared to girls. The average educational level and occupation of girls' fathers were higher than those of boys' fathers. The results also indicated significant differences between only children and non-only children in terms of age, SES, parents' educational level, and parents' occupation. Only children had a significantly higher family SES than non-only children. Furthermore, the educational level and occupation scores of parents of only children were significantly higher than those of non-only children. However, no significant difference was found in self-assessed economic status between only children and non-only children.

**Table 1:** Gender differences of participants at baseline (*N* = 3,206).

	**Gender (Mean** ±**SD)**		***t***	***p***
	**Boys (*****n*** = **1,510)**	**Girls (*****n*** = **1,696)**		
Age	13.62 ± 1.69	14.09 ± 1.85	−7.641	0.000^**^
Socioeconomic status	22.22 ± 8.87	22.86 ± 8.37	−2.088	0.037^*^
Father's education	4.61 ± 2.06	4.78 ± 2.01	−2.364	0.018^*^
Mother's education	4.45 ± 2.14	4.51 ± 2.01	−0.813	0.416
Father's job	5.26 ± 3.36	5.61 ± 3.36	−2.930	0.003^**^
Mother's job	4.88 ± 3.21	4.96 ± 3.02	−0.714	0.475
Self-assessed economic status	3.02 ± 0.68	3.00 ± 0.54	0.887	0.375
Social skills	119.74 ± 28.02	122.14 ± 24.80	−2.548	0.011^*^
Antisocial behavior	48.76 ± 19.89	43.32 ± 14.95	8.661	0.000^**^
Skipping rope	2.65 ± 1.47	2.65 ± 1.46	0.029	0.977
Roller skating	1.85 ± 1.32	1.62 ± 1.13	5.235	0.000^**^
Tag games	2.23 ± 1.39	2.01 ± 1.36	4.376	0.000^**^
Walking	3.23 ± 1.46	3.23 ± 1.43	−0.115	0.908
Cycling	2.58 ± 1.56	2.22 ± 1.43	6.676	0.000^**^
Jogging	3.36 ± 1.33	3.36 ± 1.25	−0.046	0.963
Other aerobic (e.g., mountain climbing)	3.06 ± 1.50	2.71 ± 1.48	6.720	0.000^**^
Swimming	1.79 ± 1.30	1.61 ± 1.18	4.113	0.000^**^
Baseball or softball	1.65 ± 1.20	1.38 ± 0.94	7.030	0.000^**^
Dancing	1.58 ± 1.13	1.92 ± 1.37	−7.633	0.000^**^
Badminton	2.38 ± 1.38	1.84 ± 1.20	11.748	0.000^**^
Skateboarding	1.75 ± 1.29	1.45 ± 1.01	7.219	0.000^**^
Football	2.14 ± 1.35	1.50 ± 1.00	15.113	0.000^**^
Volleyball	1.84 ± 1.29	1.63 ± 1.12	4.893	0.000^**^
Basketball	2.41 ± 1.39	1.73 ± 1.19	14.722	0.000^**^
Ice skating	1.64 ± 1.21	1.38 ± 0.92	6.664	0.000^**^
Skiing	1.56 ± 1.14	1.31 ± 0.84	6.911	0.000^**^

^*^p < 0.05; ^**^p < 0.01.

**Table 2:** Only child status differences of participants at baseline (*N* = 3,206).

	**Only child status (Mean** ±**SD)**		***t***	***p***
	**Only children (*****n*** = **1,339)**	**Non-only children (*****n*** = **1,867)**		
Age	13.76 ± 1.95	13.95 ± 1.66	−2.898	0.004^**^
Socioeconomic status	25.10 ± 8.93	20.73 ± 7.89	14.334	0.000^**^
Father's education	5.18 ± 2.14	4.35 ± 1.88	11.326	0.000^**^
Mother's education	5.06 ± 2.10	4.08 ± 1.95	13.491	0.000^**^
Father's job	6.08 ± 3.48	4.99 ± 3.21	9.029	0.000^**^
Mother's job	5.80 ± 3.26	4.30 ± 2.84	13.567	0.000^**^
Self-assessed economic status	2.99 ± 0.65	3.02 ± 0.58	−1.496	0.135
Social skills	122.01 ± 27.35	120.29 ± 25.65	1.802	0.072
Antisocial behavior	48.39 ± 20.72	44.08 ± 14.83	6.514	0.000^**^
Skipping rope	2.72 ± 1.40	2.60 ± 1.52	2.247	0.025^*^
Roller skating	1.71 ± 1.21	1.73 ± 1.24	−0.485	0.627
Tag games	1.98 ± 1.28	2.21 ± 1.44	−4.814	0.000^**^
Walking	3.05 ± 1.49	3.35 ± 1.40	−5.788	0.000^**^
Cycling	2.14 ± 1.34	2.57 ± 1.58	−8.236	0.000^**^
Jogging	3.21 ± 1.29	3.46 ± 1.28	−5.458	0.000^**^
Other aerobic (e.g., mountain climbing)	2.73 ± 1.47	2.98 ± 1.51	−4.795	0.000^**^
Swimming	1.68 ± 1.17	1.70 ± 1.28	−0.608	0.543
Baseball or softball	1.60 ± 1.11	1.44 ± 1.05	4.138	0.000^**^
Dancing	1.72 ± 1.18	1.79 ± 1.34	−1.403	0.161
Badminton	2.02 ± 1.24	2.14 ± 1.36	−2.691	0.007^**^
Skateboarding	1.62 ± 1.17	1.57 ± 1.16	1.163	0.245
Football	1.78 ± 1.17	1.82 ± 1.25	−0.964	0.335
Volleyball	1.87 ± 1.28	1.62 ± 1.14	5.742	0.000^**^
Basketball	2.05 ± 1.31	2.06 ± 1.35	−0.200	0.842
Ice skating	1.54 ± 1.10	1.48 ± 1.05	1.599	0.110
Skiing	1.47 ± 1.03	1.40 ± 0.98	1.884	0.060

^*^p < 0.05; ^**^p < 0.01.

### 3.2 Physical activity, social skills, and antisocial behavior

As shown in Tables 1, 2, using the first wave of data (2021–2022) as an example, we conducted a differential analysis of all students' participation in different types of PA, social skills, and ASB.

**Figure 1:**
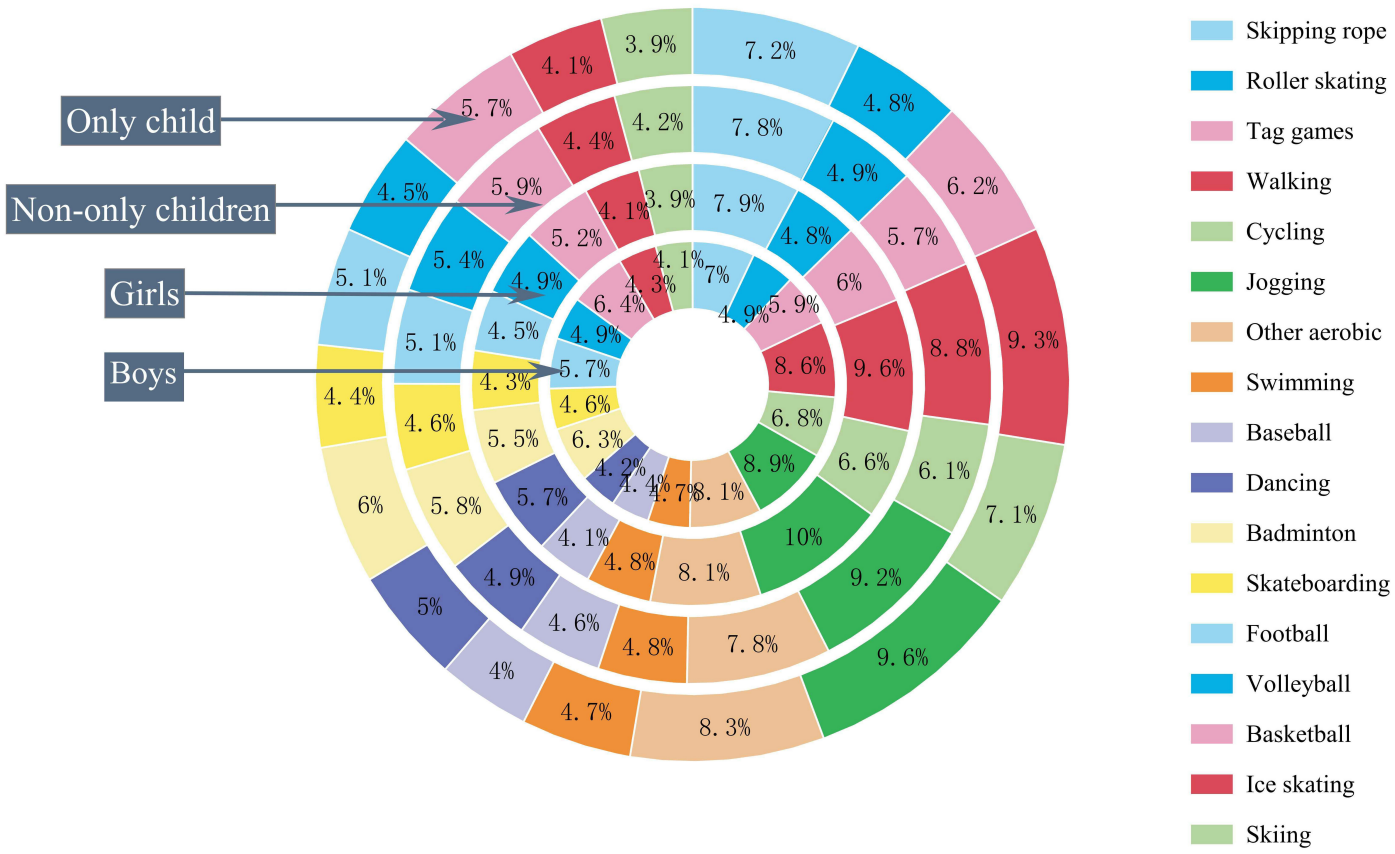
Gender and only child status differences in physical activity.

The results showed that the most frequently participated activities among adolescents were walking and jogging, followed by cycling, skipping rope, and other aerobic exercises (shown in Figure 1). There were significant gender differences in the frequency of participation in some types of PA. Boys had significantly higher participation rates in roller skating, tag games, cycling, other aerobic exercises, baseball or softball, badminton, skateboarding, football, basketball, volleyball, ice skating, and skiing compared to girls. Girls only had a significantly higher participation rate in dance. There were no significant gender differences in skipping rope, walking, and jogging. This suggests that boys have a higher frequency of PA participation per week compared to girls.

There were also differences in the frequency of PA participation between only children and non-only children. We observed that only children had a higher frequency of skipping rope, baseball or softball, and volleyball participation per week, while non-only children had higher frequencies of walking, cycling, jogging, other aerobic exercises, and badminton.

Additionally, we found that girls' social skills were significantly higher than boys', but boys' ASB was significantly higher than girls'. ASB was significantly higher among only children compared to non-only children, while there was no difference in social skills based on only-child status.

### 3.3 The relationship of physical activity, social skills, and antisocial behavior

We used FE to examine the impact of the frequency of participation in different types of PA on adolescents' social skills and ASB (shown in Table 3). Model 1 showed the effect of PA on social skills, while Model 2 added control variables to the original model. Model 3 displayed the effect of PA on ASB, and Model 4 added control variables to the original model.

**Table 3:** Physical activity, social skills, antisocial behavior among participants.

	**Model 1**		**Model 2**		**Model 3**		**Model 4**	
	**Social skills**		**Social skills**		**Antisocial behavior**		**Antisocial behavior**	
	β	**t**	β	**t**	β	**t**	β	**t**
Skipping rope	2.264^***^	5.65	2.284^***^	5.76	−1.536^***^	−5.63	−1.596^***^	−5.86
Roller skating	0.529	0.85	0.566	0.91	0.546	1.26	0.546	1.25
Tag games	−1.649^***^	−3.88	−1.615^***^	−3.83	1.655^***^	5.44	1.685^***^	5.54
Walking	3.682^***^	10.02	3.495^***^	9.53	−1.744^***^	−7.17	−1.814^***^	−7.44
Cycling	1.399^***^	3.52	1.271^***^	3.21	−1.074^***^	−4.11	−1.066^***^	−4.07
Jogging	2.019^***^	4.81	2.614^***^	5.92	−1.672^***^	−5.82	−1.617^***^	−5.30
Other aerobic	0.507	1.34	0.664^*^	1.77	0.108	0.42	0.173	0.68
Swimming	−2.633^***^	−4.10	−2.862^***^	−4.42	1.110^**^	2.42	0.947^**^	2.06
Baseball	−0.245	−0.34	−0.198	−0.27	0.225	0.41	0.263	0.49
Dancing	−1.067^**^	−2.21	−1.110^**^	−2.29	0.609^*^	1.66	0.521	1.41
Badminton	1.357^***^	2.84	1.409^***^	2.96	−0.268	−0.80	−0.220	−0.65
Skateboarding	−0.124	−0.18	−0.227	−0.33	−0.753	−1.53	−0.717	−1.46
Football	−0.678	−1.10	−0.529	−0.86	0.355	0.82	0.373	0.86
Volleyball	0.441	0.83	0.318	0.60	0.526	1.36	0.471	1.22
Basketball	0.623	1.34	0.631	1.36	−0.016	−0.05	0.017	0.05
Ice skating	0.816	0.98	0.809	0.97	1.830^***^	2.79	1.772^***^	2.71
Skiing	−2.709^***^	−3.23	−2.771^***^	−3.27	1.511^**^	2.35	1.468^**^	2.31
Age			1.780^***^	2.99			−0.623	−1.49
Socioeconomic status			0.253^***^	4.38			0.162^***^	3.72
Constant	100.821^***^	74.09	68.348^***^	7.47	50.823^***^	49.46	56.354^***^	8.85
Observations	6,412	6,412	6,412	6,412	6,412	6,412	6,412	6,412
N	3,206	3,206	3,206	3,206	3,206	3,206	3,206	3,206
R2	0.141	0.141	0.149	0.149	0.123	0.123	0.128	0.128
Adj. R2	0.138	0.138	0.146	0.146	0.121	0.121	0.126	0.126
F	30.81	30.81	28.98	28.98	21.22	21.22	20.21	20.21

^***^p < 0.01; ^**^p < 0.05; ^*^p < 0.1.

The results indicated that, after controlling for some variables, skipping rope (β = 2.284, t = 5.76), walking (β = 3.495, t = 9.53), cycling (β = 1.271, t = 3.21), jogging (β = 2.614, t = 5.92), and badminton (β = 1.409, t = 2.96) had a positive impact on adolescents' social skills. Tag games (β = −1.615, t = −3.83), swimming (β = −2.862, t = −4.42), dancing (β = −1.11, t = −2.29), and skiing (β = −2.771, t = −3.27) had a negative impact on adolescents' social skills. Skipping rope (β = −1.596, t = −5.86), walking (β = −1.814, t = −7.44), cycling (β = −1.066, t = −4.07), and jogging (β = −1.617, t = −5.30) tended to reduce adolescents' ASB. Tag games (β = 1.685, t = 5.54), swimming (β = 0.947, t = 2.06), ice skating (β = 1.772, t = 2.71), and skiing (β = 1.468, t = 2.31) tended to increase adolescents' ASB.

These findings suggest that certain types of PA can have both positive and negative effects on adolescents' social skills and ASB, and that the impact can be influenced by the inclusion of control variables in the model.

### 3.4 Difference analysis

#### 3.4.1 The relationship between physical activity and social skills

In this study, we used FE to conduct heterogeneity analysis of the relationship between different types of PA and social skills among adolescents, based on only-child status and gender. The results are presented in Table 4. Models 1 and 2 show the differences between genders and whether the child is an only child, while Models 3 and 4 add control variables to the original models.

**Table 4:** The relationship between physical activity and social skills.

	**Model 1**		**Model 2**		**Model 3**		**Model 4**	
	**Boys** β **(t)**	**Girls** β **(t)**	**Only children** β **(t)**	**Non–only children** β **(t)**	**Boys** β **(t)**	**Girls** β **(t)**	**Only children** β **(t)**	**Non-only children** β **(t)**
Skipping rope	3.182^***^	1.493^***^	2.370^***^	1.880^***^	3.192^***^	1.416^**^	2.251^***^	1.891^***^
	(5.46)	(2.69)	(3.77)	(3.53)	(5.57)	(2.55)	(3.60)	(3.58)
Roller skating	1.005	−0.054	−0.877	1.604^**^	1.069	−0.034	−1.002	1.794^**^
	(1.18)	(−0.06)	(−0.87)	(1.98)	(1.25)	(−0.04)	(−0.99)	(2.23)
Tag games	−1.694^***^	−1.329^**^	−1.647^**^	−1.502^***^	−1.591^***^	−1.373^**^	−1.530^**^	−1.496^***^
	(−2.77)	(−2.22)	(−2.34)	(−2.76)	(−2.63)	(−2.31)	(−2.20)	(-2.76)
Walking	3.297^***^	4.194^***^	4.102^***^	3.924^***^	2.983^***^	4.110^***^	3.967^***^	3.756^***^
	(6.14)	(8.31)	(7.47)	(7.87)	(5.55)	(8.21)	(7.17)	(7.62)
Cycling	1.749^***^	1.045^*^	1.303^**^	1.610^***^	1.623^***^	0.953^*^	1.348^**^	1.494^***^
	(3.09)	(1.88)	(2.06)	(3.23)	(2.88)	(1.72)	(2.12)	(3.02)
Jogging	2.876^***^	1.428^**^	4.187^***^	0.374	3.570^***^	1.853^***^	4.253^***^	1.266^**^
	(4.62)	(2.51)	(6.54)	(0.68)	(5.46)	(3.10)	(6.51)	(2.13)
Other aerobic	1.081^*^	−0.184	1.131^*^	0.096	1.336^**^	−0.117	1.324^**^	0.295
	(1.94)	(−0.36)	(1.93)	(0.20)	(2.38)	(−0.23)	(2.28)	(0.61)
Swimming	−2.167^**^	−2.678^***^	−1.872^*^	−2.641^***^	−2.427^**^	−2.893^***^	−2.062^*^	−2.882^***^
	(−2.20)	(−3.20)	(−1.68)	(−3.34)	(−2.44)	(−3.43)	(−1.85)	(-3.62)
Baseball	−1.046	0.263	−1.695	0.228	−0.933	0.357	−1.581	0.137
	(−1.06)	(0.24)	(−1.55)	(0.24)	(−0.93)	(0.32)	(−1.43)	(0.14)
Dancing	−0.531	−0.926	−0.968	−1.144^*^	−0.856	−0.845	−1.116	−1.119^*^
	(−0.64)	(−1.51)	(−1.28)	(−1.74)	(−1.03)	(−1.35)	(−1.48)	(-1.67)
Badminton	0.989	1.532^**^	1.554^**^	1.098^*^	1.136	1.473^**^	1.625^**^	1.125^*^
	(1.41)	(2.39)	(2.05)	(1.80)	(1.63)	(2.30)	(2.16)	(1.85)
Skateboarding	−1.261	0.835	−0.672	−0.070	−1.486	0.927	−0.648	−0.231
	(−1.28)	(0.86)	(−0.62)	(−0.08)	(−1.51)	(0.95)	(−0.60)	(-0.26)
Football	−1.479^*^	0.121	0.504	−1.222^*^	−1.197	0.119	0.599	−1.020
	(−1.82)	(0.13)	(0.49)	(−1.67)	(−1.48)	(0.12)	(0.58)	(-1.40)
Volleyball	0.235	0.826	−0.527	0.833	0.161	0.629	−0.664	0.606
	(0.30)	(1.13)	(−0.74)	(1.06)	(0.21)	(0.87)	(−0.94)	(0.78)
Basketball	0.246	0.660	−0.528	1.358^**^	0.248	0.720	−0.421	1.330^**^
	(0.37)	(0.97)	(−0.71)	(2.24)	(0.37)	(1.07)	(−0.57)	(2.20)
Ice skating	1.281	0.306	−1.373	1.829^*^	1.282	0.283	−1.482	1.963^*^
	(1.06)	(0.27)	(−1.06)	(1.77)	(1.07)	(0.25)	(−1.14)	(1.88)
Skiing	−2.341^**^	−3.426^**^	0.070	−3.640^***^	−2.314^**^	−3.557^***^	−0.143	−3.683^***^
	(−2.22)	(−2.56)	(0.05)	(−3.65)	(−2.19)	(−2.61)	(−0.10)	(-3.64)
Age					2.227^**^	1.058	−0.334	2.840^***^
					(2.43)	(1.31)	(−0.37)	(3.53)
Socioeconomic status					0.243^***^	0.319^***^	0.246^***^	0.219^***^
					(2.84)	(4.14)	(2.84)	(2.81)
Constant	96.067^***^	104.383^***^	96.884^***^	102.801^***^	57.535^***^	81.260^***^	95.967^***^	54.788^***^
	(45.24)	(59.29)	(42.85)	(60.77)	(4.14)	(6.52)	(6.90)	(4.43)
Observations	3,020	3,392	2,678	3,734	3,020	3,392	2,678	3,734
N	1,510	1,696	1,339	1,867	1,510	1,696	1,339	1,867
R2	0.165	0.129	0.206	0.125	0.174	0.140	0.212	0.136
Adj. R2	0.161	0.125	0.201	0.121	0.168	0.135	0.206	0.132
F	18.50	14.48	21.11	14.98	17.37	14.26	19.12	14.51

^***^p < 0.01; ^**^p < 0.05; ^*^p < 0.1. Robust t-statistics in parentheses.

Regarding gender differences, badminton only had a positive effect on girls' social skills (Girls: β = 1.473, t = 2.30), but no significant effect on boys. Other aerobic exercises only had a significant positive effect on boys (Boys: β = 1.336, t = 2.38), with no effect on girls. Additionally, skipping rope (Boys: β = 3.192, t = 5.57; Girls: β = 1.416, t = 2.55), walking (Boys: β = 2.983, t = 5.55; Girls: β = 4.110, t = 8.21), cycling (Boys: β = 1.623, t = 2.88; Girls: β = 0.953, t = 1.72), and jogging (Boys: β = 3.570, t = 5.46; Girls: β = 1.853, t = 3.10) had a positive effect on the social skills of both boys and girls, with no gender differences. Tag games (Boys: β = −1.591, t = −2.63; Girls: β = −1.373, t = −2.31), swimming (Boys: β = −2.427, t = −2.44; Girls: β = −2.893, t = −3.43), and skiing (Boys: β = −2.314, t = −2.19; Girls: β = −3.557, t = −2.61) had a significant negative effect on both boys and girls.

Regarding the differences in only-child status, roller skating, other aerobic exercises, dancing, basketball, ice skating, and skiing differences based on whether the child is an only child. Non–only children who participated in roller skating (Non–only children: β = 1.794, t = 2.23), basketball (Non–only children: β = 1.330, t = 2.20), and ice skating (Non–only children: β = 1.963, t = 1.88) had improved social skills, while no effect was found for only children. Dancing (Non–only children: β = −1.119, t = −1.67) and skiing (Non–only children: β = −3.683, t = −3.64) reduced the social skills of non–only children, but no significant effect was found for only children. Participation in other aerobic exercises (Only Children: β = 1.324, t = 2.28) improved the social skills of only children, with no effect on non–only children. Additionally, skipping rope (Only Children: β = 2.251, t = 3.60; Non–only children: β = 1.891, t = 3.58), tag games (Only Children: β = −1.530, t = −2.20; Non–only children: β = −1.496, t = −2.76), walking (Only Children: β = 3.967, t = 7.17; Non–only children: β = 3.756, t = 7.62), cycling (Only Children: β = 1.348, t = 2.12; Non–only children: β = 1.494, t = 3.02), jogging (Only Children: β = 4.253, t = 6.51; Non–only children: β = 1.266, t = 2.13), swimming (Only Children: β = −2.062, t = −1.85; Non–only children: β = −2.882, t = −3.62), and badminton (Only Children: β = 1.625, t = 2.16; Non–only children: β = 1.125, t = 1.85) had a significant effect on the social skills of both only children and non–only children, with no gender differences.

#### 3.4.2 The relationship between physical activity and antisocial behavior

We conducted a heterogeneity analysis using the FE to examine the relationship between different types of PA and ASB among adolescents, based on only-child status and gender. The results are presented in Table 5. Models 1 and 2 show the differences between genders and whether the child is an only child, while Models 3 and 4 add control variables to the original models.

**Table 5:** The relationship between physical activity and antisocial behavior.

	**Model 1**		**Model 2**		**Model 3**		**Model 4**	
	**Boys** β **(t)**	**Girls** β **(t)**	**Only children** β **(t)**	**Non-only children** β **(t)**	**Boys** β **(t)**	**Girls** β **(t)**	**Only children** β **(t)**	**Non-only children** β **(t)**
Skipping rope	−2.246^***^	−0.819^**^	−1.498^***^	−1.291^***^	−2.219^***^	−0.896^**^	−1.672^***^	−1.295^***^
		(−5.43)	(−2.29)	(−3.13)	(−3.94)	(−5.37)	(−2.51)	(−3.50)	(−3.94)
Roller skating	0.870	0.067	1.287^*^	0.174	0.839	0.079	1.197	0.234
		(1.41)	(0.11)	(1.74)	(0.33)	(1.35)	(0.13)	(1.63)	(0.45)
Tag games	1.979^***^	1.112^***^	1.484^***^	1.734^***^	2.006^***^	1.092^**^	1.602^***^	1.731^***^
		(4.47)	(2.61)	(2.77)	(4.87)	(4.57)	(2.57)	(3.00)	(4.85)
Walking	−2.027^***^	−1.522^***^	−2.679^***^	−1.247^***^	−2.034^***^	−1.569^***^	−2.746^***^	−1.309^***^
		(−5.25)	(−4.97)	(−6.84)	(−3.90)	(−5.23)	(−5.11)	(−6.98)	(−4.10)
Cycling	−1.205^***^	−0.899^**^	−1.472^***^	−0.802^***^	−1.174^***^	−0.932^***^	−1.341^***^	−0.840^***^
		(−3.09)	(−2.57)	(−3.18)	(−2.67)	(−3.03)	(−2.65)	(−2.85)	(−2.80)
Jogging	−1.991^***^	−1.270^***^	−2.207^***^	−1.319^***^	−2.070^***^	−1.078^***^	−2.302^***^	−1.079^***^
		(−4.23)	(−3.62)	(−4.76)	(−3.66)	(−4.11)	(−2.97)	(−4.76)	(−2.82)
Other aerobic	0.034	0.141	0.135	0.028	0.036	0.185	0.312	0.073
		(0.08)	(0.44)	(0.31)	(0.09)	(0.09)	(0.57)	(0.70)	(0.24)
Swimming	0.801	1.525^**^	0.916	1.383^***^	0.698	1.387^**^	0.765	1.264^**^
		(1.15)	(2.46)	(0.98)	(2.79)	(1.00)	(2.23)	(0.82)	(2.55)
Baseball	0.261	0.294	0.303	0.484	0.230	0.351	0.352	0.471
		(0.34)	(0.35)	(0.34)	(0.71)	(0.30)	(0.43)	(0.41)	(0.69)
Dancing	0.768	0.181	2.139^***^	−0.411	0.810	0.190	1.988^***^	−0.445
		(1.08)	(0.44)	(3.34)	(−0.93)	(1.14)	(0.44)	(3.10)	(−1.01)
Badminton	−0.570	0.154	−1.086^*^	0.168	−0.548	0.133	−1.022^*^	0.193
		(−1.10)	(0.36)	(−1.87)	(0.41)	(−1.06)	(0.31)	(−1.76)	(0.47)
Skateboarding	−1.784^**^	0.485	−1.181	−0.562	−1.762^**^	0.558	−1.123	−0.582
		(−2.53)	(0.71)	(−1.33)	(−1.03)	(−2.49)	(0.83)	(−1.27)	(−1.07)
Football	1.544^***^	−1.000	0.055	0.396	1.405^**^	−0.983	0.116	0.438
		(2.63)	(−1.48)	(0.07)	(0.80)	(2.40)	(−1.45)	(0.15)	(0.89)
Volleyball	0.150	0.658	1.691^***^	−0.390	0.206	0.535	1.549^***^	−0.430
		(0.24)	(1.35)	(3.09)	(−0.72)	(0.34)	(1.09)	(2.81)	(−0.79)
Basketball	−0.168	0.358	0.275	−0.228	−0.205	0.410	0.361	−0.242
		(−0.35)	(0.76)	(0.47)	(−0.57)	(−0.43)	(0.86)	(0.61)	(−0.60)
Ice skating	2.897^***^	0.677	3.030^***^	1.179	2.834^***^	0.641	2.928^***^	1.190
		(3.09)	(0.75)	(2.90)	(1.41)	(3.03)	(0.72)	(2.81)	(1.42)
Skiing	1.108	2.490^**^	0.926	1.509^*^	1.046	2.417^**^	0.722	1.514^*^
		(1.31)	(2.55)	(0.82)	(1.93)	(1.24)	(2.51)	(0.65)	(1.95)
Age					−1.509^**^	0.268	−1.214^*^	0.375
						(−2.26)	(0.48)	(−1.69)	(0.76)
Socioeconomic status					0.108	0.199^***^	0.197^***^	0.109^**^
						(1.57)	(3.73)	(2.84)	(2.00)
Constant	56.047^***^	45.526^***^	53.332^***^	48.982^***^	75.516^***^	37.015^***^	66.477^***^	40.784^***^
		(35.57)	(32.90)	(28.60)	(43.49)	(7.62)	(4.31)	(6.09)	(5.43)
Observations	3,020	3,392	2,678	3,734	3,020	3,392	2,678	3,734
N	1,510	1,696	1,339	1,867	1,510	1,696	1,339	1,867
R2	0.152	0.112	0.180	0.096	0.157	0.121	0.188	0.099
Adj. R2	0.147	0.108	0.175	0.0920	0.151	0.116	0.182	0.0945
F	14.11	8.736	15.37	9.274	13.51	8.513	14.67	8.653

^***^p < 0.01; ^**^p < 0.05; ^*^p < 0.1. Robust t-statistics in parentheses.

We found that the relationship between PA and ASB varied by gender. Swimming, skateboarding, football, ice skating, and skiing had significant gender differences in their impact on ASB. Specifically, swimming (Girls: β = 1.387, t = 2.23) and skiing (Girls: β = 2.417, t = 2.51) tended to increase girls' ASB, with no effect on boys. Skateboarding (Boys: β = −1.762, t = −2.49) tended to decrease boys' ASB, with no effect on girls. Football (Boys: β = 1.405, t = 2.40) and ice skating (Boys: β = 2.834, t = 3.03) tended to increase boys' ASB, with no effect on girls. Additionally, skipping rope (Boys: β = −2.219, t = −5.37; Girls: β = −0.896, t = −2.51), walking (Boys: β = −2.034, t = −5.23; Girls: β = −1.569, t = −5.11), cycling (Boys: β = −1.174, t = −3.03; Girls: β = −0.932, t = −2.65), and jogging (Boys: β = −2.070, t = −4.11; Girls: β = −1.078, t = −2.97) tended to reduce ASB in both boys and girls, with no gender differences.

We also found that swimming, dancing, badminton, volleyball, ice skating, and cross–country skiing had different effects on ASB based on only–child status. Specifically, swimming (Non–only children: β = 1.264, t = 2.55) and skiing (Non–only children: β = 1.514, t = 1.95) tended to increase ASB in non–only children, with no significant effect on only children. Dancing (Only Children: β = 1.988, t = 3.10), volleyball (Only Children: β = 1.549, t = 2.81), and ice skating (Only Children: β = 2.928, t = 2.81) tended to increase ASB in only children, with no significant effect on non–only children. Badminton (Only Children: β = −1.022, t = −1.76) tended to decrease ASB in only children, with no effect on non–only children. Furthermore, skipping rope (Only Children: β = −1.672, t = −3.50; Non–only children: β = −1.295, t = −3.94), walking (Only Children: β = −2.746, t = −6.98; Non–only children: β = −1.309, t = −4.10), cycling (Only Children: β = −1.341, t = −2.85; Non–only children: β = −0.840, t = −2.80), and jogging (Only Children: β = −2.302, t = −4.76; Non–only children: β = −1.079, t = −2.82) tended to reduce ASB in both only children and non–only children.

## 4 Discussion

This study conducted a two-year longitudinal survey of Chinese adolescents, using FE to investigate the impact of PA on social skills and ASB in 1,510 boys and 1,696 girls, and conducted a heterogeneity analysis based on only-child status and gender. Our research findings are as follows:

(1) Not all PA improved the social skills of Chinese adolescents or reduced their ASB. The relationship between PA and these outcomes varied by gender and only-child status. (2) Boys had significantly higher participation rates in PA such as roller skating, tag games, cycling, other aerobic exercises, baseball or softball, badminton, skateboarding, football, basketball, volleyball, ice skating, and skiing compared to girls, with the exception of dancing, where girls had a significantly higher participation rate. There were no significant gender differences in skipping rope, walking, and jogging. (3) Only children had higher participation rates in skipping rope, baseball or softball, and volleyball compared to non-only children, but non-only children had higher participation rates in walking, cycling, jogging, other aerobic exercises, and badminton. There were no significant differences in participation rates in roller skating, swimming, dancing, skateboarding, football, basketball, ice skating, and skiing based on only-child status. (4) Girls had higher social skills, but boys had significantly higher rates of ASB. (5) Only children had significantly higher rates of ASB compared to non-only children, while there was no difference in social skills based on only-child status.

PA is widely recognized as influencing adolescents' social skills and ASB. Participation in PA can enhance social and emotional development, fostering the ability to handle relationships with oneself, others, and the community (35, 36). Engaging in PA promotes better social skills among adolescents (37). At the same time, adolescents with psychosocial problems are also associated with lower levels of PA participation (38), leading to a potential vicious cycle. Therefore, we believe that society, schools, and teachers should take further measures to promote PA among students. However, it is worth noting that some scholars have reported inconsistent results, suggesting that PA indices are unrelated to social and emotional factors (39). These inconsistencies may arise from the variety of PA, as different activities have different health outcomes. Therefore, we further explored the impact of different types of PA on social skills and ASB.

The findings indicate that skipping rope, walking, cycling, jogging, and badminton have a positive impact on adolescents' social skills, while tag games, swimming, dancing, and skiing have a negative impact. Conversely, skipping rope, walking, cycling, and jogging can reduce ASB, whereas tag games, swimming, ice skating, and skiing can increase it. A possible explanation for this pattern is that activities like skipping rope, walking, cycling, and jogging are more structured and are often part of daily routines or school physical education, while tag games, swimming, ice skating, and skiing are unstructured leisure time PA (40). Participation in structured activities is linked to lower levels of ASB, while participation in unstructured activities is associated with higher levels of ASB (41). Therefore, we suggest that adolescents should engage in more structured PA as part of their daily routine or in school settings.

Interestingly, we also found demographic differences in the impact of PA. For social skills, badminton only had a positive effect on girls, while other aerobic activities had a significant positive effect on boys. Non-only children's participation in roller skating, basketball, and ice skating was beneficial for their social skills, while dancing and skiing reduced social skills. Additionally, participation in other aerobic activities improved the social skills of only children. Regarding ASB, swimming and skiing were found to increase ASB in girls and only children. Skateboarding reduced ASB in boys, while football and ice skating increased it. Dancing, volleyball, and ice skating increased ASB in only children, while badminton reduced it. Moreover, skipping rope, walking, cycling, and jogging were found to reduce ASB and improve social skills without demographic differences.

From the perspective of differential analysis, the reasons for these differences may stem from the different environments in which PA are participated in. For example, boys may dislike structured exercise classes the most, while girls dislike completely solitary exercise environments the most (42). Swimming, being a solitary activity, may lead to a rejection of the sport due to the exercise environment and lacks a social context, which is not conducive to social behavior. In addition to the exercise environment, the intensity of the exercise is also a factor, as research has shown that vigorous PA can increase criminal behavior in males (43). Other studies have shown the opposite, that PA can reduce the risk of bullying for males (44). Another potential factor is the significant differences in sensitivity, ASB, and social emotional issues among boys and girls, and between only children and non-only children, which contribute to the marked inconsistencies (45–48). While gender differences are well-documented, the differences between only children and non-only children provide new evidence and a unique perspective for related research.

Our findings support previous research on gender differences in PA. Despite the higher participation of girls in dancing and no significant gender differences in jogging, skipping rope, and walking, overall PA was lower among girls (45, 49–51). This discrepancy is reflected in activities like roller skating, tag games, cycling, other aerobic exercises, baseball or softball, badminton, skateboarding, football, basketball, volleyball, ice skating, and skiing. We suggest that psychological enjoyment and cultural background may play roles in these differences (52). Boys may have a stronger preference for PA due to their greater physical capabilities (53), while girls may have a higher innate talent and preference for dance. Girls may also experience negative emotions due to internalized behaviors, such as dissatisfaction with their body shape or athletic ability (46), and may lack the motivation to engage in PA due to a lack of enjoyment, social support, and past experience (47). Overall, girls may have a more complex emotional landscape and greater barriers to PA participation compared to boys (45, 47).

PA preferences also varied between only children and non-only children. Non-only children had higher participation rates in walking, cycling, jogging, other aerobic exercises, and badminton. These activities are often categorized as daily PA due to their universality, convenience, and widespread availability. Therefore, it can be seen that the participation of non-only children in sports activities in daily life is higher than that of only children (54, 55). This is consistent with studies by scholars that non-only children have higher levels of moderate and vigorous PA (31). However, only children had higher participation rates in skipping rope, baseball or softball, and volleyball. This could be due to parental support (56), as these activities may not be as universally accessible and could be influenced by limited attention theory (57). Additionally, skipping rope is a physical education requirement in China's compulsory education system, which may also contribute to higher participation rates among only children.

The study revealed that girls had higher social skills than boys, but boys exhibited significantly higher rates of ASB. This aligns with some scholars' findings that girls have better environmental adaptability and social skills (58). According to social role theory, girls may appear warmer than boys (59). Girls tend to excel in social behavior and social responsibility compared to boys (60). However, despite having stronger interpersonal relationship skills, girls often experience more stress and negative emotions due to their sensitivity (21), which can lead to poorer psychological wellbeing than boys. In terms of ASB, boys show more overt behaviors such as aggression, truancy, smoking, and alcohol consumption (61). Boys' social cognitive style, status, and agentic goals, as well as their information processing methods, contribute to their higher rates of aggression (21, 48). Therefore, parents and teachers should pay more attention to the ASB of adolescent boys, enhancing their social behavior and interpersonal communication skills. At the same time, attention should be given to the internalizing behaviors of adolescent girls to prevent psychological disorders or negative health outcomes due to social pressures and negative emotions.

Additionally, the study found that only children had significantly higher rates of ASB than non-only children, while there was no difference in social skills based on only-child status. Contrary to our findings, some scholars have suggested that non-only children are more prone to engaging in aggressive behaviors such as fighting, self-harm, and suicidal ideation than only children (62). Potential explanations for these discrepancies may lie in the different family structures, family environments, and access to human and material resources for only child and non-only children (28). Family structure and environment are complex factors strongly associated with adolescents' ASB (63, 64). Moreover, parents' social skills play a crucial role in how children interact with their peers at school (65). Therefore, we believe that regardless of whether they are only child or not, parents should provide a healthy family environment, pay attention to their social skills and ASB to promote their healthy development (66).

The study aims to draw attention to the potential of PA as a tool for interventions targeting adolescent social behavior. Our results provide a novel understanding that can inform interventions and improvements in social behavior among Chinese adolescents. By recognizing the differential impacts of PA on social skills and ASB, stakeholders such as educators, policymakers, and health professionals can develop more tailored and effective strategies to promote positive social development among adolescents. This could include the design of PA programs that are sensitive to gender and family structure, as well as the incorporation of activities that have been shown to positively influence social skills and reduce ASB in different demographic groups.

### 4.1 Limitation

Despite the contributions of this study to the understanding of PA, social skills, and ASB among Chinese adolescents, and the consideration of gender and only-child status in the differential analysis, it is important to acknowledge its limitations.

Firstly, beyond SES, gender age, and only-child status, there are many potential confounding factors that were not included in this study. For instance, the stigmatization of overweight and obesity can have a detrimental impact on adolescents' participation in PA and social interaction, which may potentially influence the outcomes. Future research should consider including obesity indices (67). Secondly, adolescents' PA is influenced by school environment and parental PA behavior. Further research should explore the complex social context of adolescents to investigate the relationship between these factors more thoroughly. Thirdly, the types of PA collected in this study were limited to those commonly practiced by Chinese adolescents, and did not cover all possible activities. A broader range of activities should be considered in future research to provide a more comprehensive picture. Fourthly, there are significant urban-rural disparities in China, which may affect the outcomes for adolescents. Future research should delve deeper into these disparities to understand their impact on adolescents' social skills and ASB. In conclusion, while this study offers valuable insights into the relationship between PA, social skills, and ASB among Chinese adolescents, there is room for improvement in terms of the range of factors considered and the depth of analysis. Future research should aim to address these limitations to provide a more robust and nuanced understanding of the complex interplay between PA and social outcomes in adolescent populations.

## 5 Conclusion

Our findings highlight the importance of considering gender and only-child status when examining the relationships between PA, social skills, and ASB. Additionally, the inconsistent results across different types of PA, as well as the gender and only-child differences observed, underscore the complex nature of these relationships. These insights are particularly significant for shaping the health and wellbeing of Chinese adolescents.

## Data Availability

The datasets presented in this study can be found in online repositories. The names of the repository/repositories and accession number(s) can be found below: Database of Youth Health (https://www.ncmi.cn/index.html).
